# New Advances in Targeted Therapy of HER2-Negative Breast Cancer

**DOI:** 10.3389/fonc.2022.828438

**Published:** 2022-03-04

**Authors:** Junsha An, Cheng Peng, Xiaofang Xie, Fu Peng

**Affiliations:** ^1^ Key Laboratory of Drug-Targeting and Drug Delivery System of the Education Ministry and Sichuan Province, Sichuan Engineering Laboratory for Plant-Sourced Drug and Sichuan Research Center for Drug Precision Industrial Technology, West China School of Pharmacy, Sichuan University, Chengdu, China; ^2^ State Key Laboratory Southwestern Chinese Medicine Resources, Chengdu University of Traditional Chinese Medicine, Chengdu, China

**Keywords:** HER2-negative, breast cancer, targeted therapy, inhibitors, multiple targets

## Abstract

Breast cancer has an extremely high incidence in women, and its morbidity and mortality rank first among female tumors. With the increasing development of molecular biology and genomics, molecular targeted therapy has become one of the most active areas in breast cancer treatment research and has also achieved remarkable achievements. However, molecular targeted therapy is mainly aimed at HER2-positive breast cancer and has not yet achieved satisfactory curative effect on HER2-negative breast cancer. This article describes the potential targets that may be used for breast cancer treatment from the aspects of PI3K/AKT signaling pathway, DDR, angiogenesis, the cell cycle, breast cancer stem cells, *etc.*, and explores possible inhibitors for the treatment of HER2-negative breast cancer, such as PI3K inhibitors, AKT inhibitors and m-TOR inhibitors that inhibit the PI3K/AKT signaling pathway, small molecule tyrosine kinase inhibitors that restrain angiogenesis, CDK inhibitors, aurora kinase inhibitors and HDAC inhibitors that block cell cycle, as well as the drugs targeting breast cancer stem cells which have been a hit, aiming to provide a new idea and strategy for the treatment of HER2-negative breast cancer.

## Background

Breast cancer is the most common female malignant tumor worldwide, and it is also the main cause of death of women from cancer. Its morbidity and mortality are still rising, and the trend is getting younger (1), so the exploration of the occurrence, development and treatment of breast cancer has long been a hot spot of global concern (2).

After gene and protein level detection, breast cancer is divided into five molecular subtypes according to the characteristics of gene and protein expression: normal-like breast cancer, luminalA, luminalB, HER2-enriched breast cancer (HER2-E) and basal-like breast cancer, namely triple negative breast cancer (TNBC) (3). HER2 expressed in HER2-enriched and part of luminalB breast cancer is the membrane receptor encoded by the proto-oncogene ERBB2, which is a member of human epidermal growth factor receptor (EGFR/ERB) family of tyrosine kinase receptors (4). HER2-negative breast cancer refers to normal-like breast cancer, luminalA, partial luminalB and basal-like type. Currently, the treatment methods for breast cancer include surgery, radiotherapy, chemotherapy, endocrine therapy and targeted therapy (5). LuminalA and luminalB respond well to hormone therapy, while HER2-E has been developed to target HER2. Basal-like drugs are highly invasive and there is currently no molecular-based targeted therapy (6). In addition, surgical resection combined with chemotherapy is still the main method for the treatment of breast cancer, but such treatment has serious side effects and serious physical and mental impact on patients. Targeted therapy has the advantages of strong specificity, significant efficacy and small side effects, and is an effective choice among many clinical treatment plans (7). For patients with HER2-positive breast cancer, several new targeted drugs have been specifically used for the treatment in the past 10 years, including the application of trastuzumab and pertuzumab, which have significantly improved the survival rate of patients, indicating that targeted therapy is a powerful means for the treatment of breast cancer (8). **Table 1** shows the five molecular subtypes, gene expression profiles and treatment strategies of breast cancer (6). It can be seen that HER2-targeted drugs are only effective for a small number of breast cancer patients and have no obvious effect on the majority of breast cancer patients. Therefore, the application of targeted drugs is still limited at present.

**Table 1:** Five molecular subtypes and related characteristics of breast cancer.

Intrinsic subtype	Gene expression profile	Proportion	Characteristic targets/markers expression	Treatment strategies
Normal-like	ER- and PR- and HER2-	5%-10%	Negative for CK5 and EGFR	Chemotherapy drugs
Luminal A	ER+ and/or PR+ and HER2-	50%-60%	CK8/18, genes associated with ER function like LIV1, FOXA1, XBP1, GATA3	Hormonal therapy and chemotherapy drugs
Luminal B	ER+ and/or PR+ and HER2-/HER2+	15%-20%	v-MYB, LAPTMB4, NSEP1, cyclinE1, Ki67, FGFR1, FGFR2, PI3K	Hormonal therapy and chemotherapy drugs
HER2-E	ER- and PR- and HER2+	15%-20%	HER2, TP53, HDPP	HER2 directed therapies
Basal-like	ER- and PR- and HER2-	8%-37%	CK5, CK14, CK17, P-cadherin, caveolins 1, EGFR, MAPK, NF-κB, Ki67, TP53, BRCA1	Chemotherapy drugs

Most of the special expression molecules of different subtypes in the table are used for breast cancer typing, such as the expression of GENES and proteins related to K67 and HR, while other molecules that are different from the expression of HER2-E can serve as potential targets for the treatment of HER2-negative breast cancer (9–11). For example, CK8/18 and GATA3, which can regulate the cell metastasis process including ZEB2, are closely related to the proliferation and metastasis of breast cancer and can be treated from the cell cycle and EMT pathway (12, 13). EGFR and FGFR1 have a regulatory effect on the generation of new blood vessels, and many angiogenesis inhibitors targeting these targets have been clinically used (14).

Exploring innovative target drugs other than HER2 and specifically targeting breast cancer with negative HER2 expression is the development trend of today’s breast cancer treatment and also one of the most challenging issues in the treatment of breast cancer (15). Therefore, we mainly provide an overview illustrating potential targets or signaling pathways using to treat breast cancer, so as to provide references for the clinical application of targeted therapy for HER2-negative breast cancer.

## PI3K/AKT Signaling Pathway

Phosphoinositide 3-kinase (PI3K)/protein kinase (AKT)/mammalian target of rapamycin (mTOR) signaling pathway, referred to as PI3K/AKT pathway, is one of the most frequently activated pathogenic signaling cascades in breast cancer (16). Abnormal activation of this pathway is the most common pathogenesis of breast cancer. It regulates survival, proliferation, differentiation, apoptosis and other processes of breast cancer cells, and performs a pivotal function in the occurrence and development (17, 18). This makes PI3K/AKT signaling pathway an important therapeutic target for research and treatment of breast cancer (19). At present, a large number of targeted drugs that act on various proteins of PI3K/AKT pathway have been developed, providing a fresh direction for the targeted therapy of HER2-negative breast cancer.

### PI3K Inhibitors

PI3K is roughly divided into type I, type II and type III, and type I is divided into type IA and IB. Among them, type I PI3K is the most widely studied, and type IA has the highest correlation with breast cancer behavior. It is the main PI3K family enzyme known to drive breast cancer (20). Studies have confirmed that the activating mutation of PIK3CA is a carcinogenic mechanism related to the excessive activation of this pathway, and this gene mutation is distributed in various breast cancer subtypes (21). Therefore, inhibiting the excessive activation of PI3K pathway makes the targeted therapy of HER2-negative breast cancer possible.

At present, PI3K inhibitors are mainly divided into three categories. The first category is pan-PI3K inhibitors, such as buparlisib and pictilisib, which can act on all different PI3K I subtypes in the mean time. Pan PI3K inhibitor is an ATP-competitive inhibitor, which has a wide range of activities by affecting a wide range of downstream targets. Meanwhile, its toxicity increases accordingly, such as hyperglycemia, anemia, neutropenia, elevated aminotransferase, rash and hepatotoxicity, *etc*. (22). The second generation inhibitors are PI3K subtype selective inhibitors, which only act on specific subtypes, and their main adverse reactions are hypertension and diarrhea. Compared with the first class of pan-PI3K inhibitors, they have the advantages of stronger efficacy, less adverse reactions and better patient tolerance (23, 24). The third-generation inhibitor is a dual PI3K-mTOR inhibitor, such as NVP-BBD130, NVP-BEZ-235, and PKI-587. These inhibitors simultaneously target two targets in PI3K/AKT pathway, enhancing better efficacy and reducing the possibility of inducing drug resistance (25).

PI3K inhibitors can be applied to a variety of breast cancer subtypes, especially breast cancer subtypes with PI3KCA mutations. On May 24, 2019, alpelisib, a selective PI3Kα inhibitor, was approved by the Food and Drug Administration (FDA) for the treatment of advanced or metastatic breast cancer patients with HR+/HER2- or PI3KCA mutations. However, the occurrence of breast cancer rarely depends solely on the PI3K signaling pathway, and PI3K inhibitors are often used in combination with other treatments to increase the sensitivity of breast cancer patients to drugs and reduce drug resistance. In addition, Guney Eskiler et al. (26) found that PI3K inhibitors can significantly inhibit the proliferation of triple-negative breast cancer (TNBC), resulting in impaired mRNA and protein expression of BRCA1 and RAD51 in cells, which in turn leads to damage to homologous recombination, and brings as a result to cell death by inducing DNA damage and promoting the overexpression of the apoptosis gene Bax.

In addition, studies have confirmed that p110γ, one of the catalytic subunits of PI3K, plays an important role in immunosuppressive function of M2-type macrophages in tumor microenvironment, and by inhibiting M2-type macrophages, PI3Kγ inhibitors can restore drug resistance of breast cancer cells to immunotherapy, such as PD-1 and CTLA-4. Therefore, the development of PI3Kγ inhibitors is expected to shift the development strategy of PI3K inhibitors from the concept of “targeted therapy” to “immunotherapy”, indicating that it has a wide application in the combined immunosuppression treatment of breast cancer (27, 28).

### AKT Inhibitors

AKT is located at the core of the PI3K/AKT pathway, regulated by various upstream signaling proteins, and then plays a role in gene transcription, protein synthesis, cell survival and proliferation through a variety of downstream pathways (29). AKT has three subtypes, AKT1, AKT2 and AKT3. They are all composed of pleckstrin homology domain (PHD), kinase domain (KD), and hydrophobic C-terminal regulatory motif (HM) (30). Among them, the activation of AKT1 can inhibit apoptosis in breast cancer cells and increase its survival rate. Therefore, inhibiting AKT in breast cancer can achieve therapeutic effects (31).

PIK3CA activation, PTEN loss mutations, and AKT1-E17K mutations are common in breast cancer, and mutations in these genes can cause AKT to be dysregulated (20, 32). The complete activation of AKT needs to be transferred to the plasma membrane. PHD can interact with PIP3 produced by the upstream kinase PIK3CA, and then AKT is recruited to the plasma membrane to play a role (33).

At present, AKT inhibitors can be divided into three main categories according to the differences in binding sites and modes of action: (1) Allosteric inhibitors, such as MK2206. Studies have shown that MK-2206 acts on most PIK3CA mutant cell lines and PTEN loss cell lines (34) and can inhibit AKT phosphorylation in platelets (35). In this regard, Yi Yu et al. have shown that ARQ 092 and ARQ 751 can inhibit the activation of AKT by actively forming and destroying the ion-membrane metastasis pathway and have a better curative effect on breast cancer cells with AKT1-E17K mutations (36). (2) PIP analogs, such as Perifosine. PIP analogs can bind to PHD, and the conjugates can’t activate AKT, and can promote breast cancer cell apoptosis. In addition, in ER-breast cancer cells, the recruitment of AKT requires the mechanism of calcium-dependent calmodulin (CaM) to occur, so CaM inhibitors can promote the apoptosis of ER-breast cancer cells (31). (3) ATP competitive inhibitors, such as ipatasertib. Ipatasertib is a highly selective ATP competitive inhibitor and the only AKT inhibitor for TNBC under clinical research (37). The high sensitivity of breast cancer cells to ipatasertib is often related to the loss of PTEN and PIK3CA mutation, which has a bright prospect in the clinical treatment of TNBC (38).

### MTOR Inhibitors

mTOR is a crucial kinase downstream of the PI3K/AKT pathway. It is composed of mTOR complex 1 (mTORC1) and mTOR complex 2 (mTORC2). It mainly regulates the growth, division and angiogenesis of breast cancer cells, and participates in the metastasis and invasion of breast cancer cells. mTORC1 is located downstream of AKT and promotes the formation of blood vessels by enhancing the transcription of proto-oncogenes to drive the formation of breast cancer. mTORC2 is located upstream of AKT and regulates the phosphorylation of AKT and cytoskeleton proteins to regulate the growth and migration (39, 40).

The first generation of mTOR inhibitors only targets mTORC1, such as everolimus, which can bind to the intracellular receptor FK506-binding protein 12 to inhibit the activity of mTOR kinase and the production of mTORC1 complex, thereby it can inhibit angiogenesis and achieve the purpose of treating breast cancer (41). However, the first generation of mTOR inhibitors has no inhibitory effect on mTORC2, which will cause negative feedback activation of AKT and its downstream pathways by RAS-MAPK, S6K1/IGF-1R/PI3K and other pathways, thereby affecting drug efficacy (42). At the same time, there may be non-infectious pneumonia, infection, mouth ulcers, kidney failure and other side effects. The second-generation mTOR inhibitors, such as AZD2014 and MLN0128, have greater therapeutic advantages than single-target inhibitors. This ATP analog inhibits the catalytic activity of mTORC1 and mTORC2 by binding to the kinase domain of mTOR. So it can reduce side effects and maximize the benefits of patients (43).

**Figure 1:**
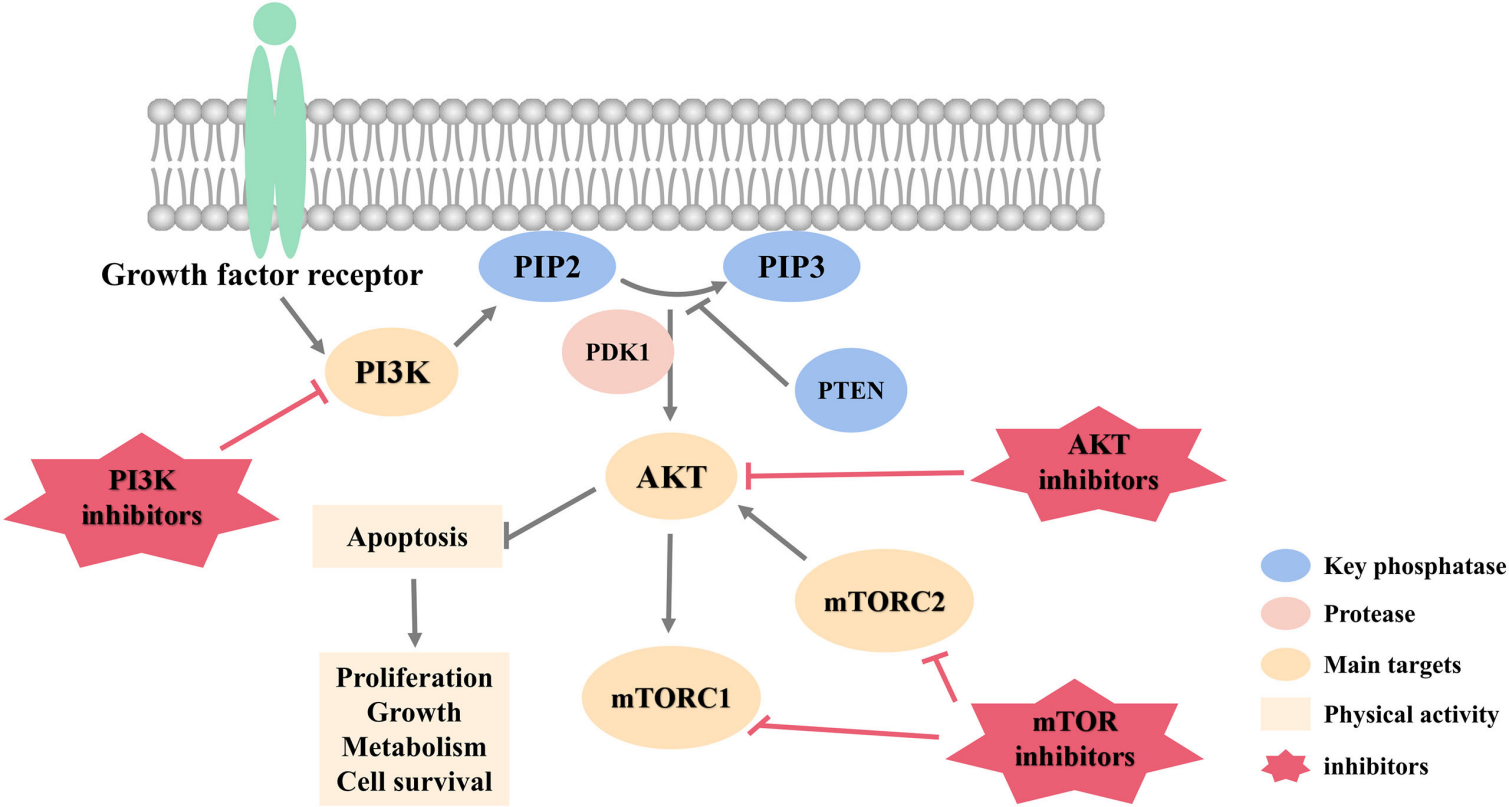
PI3K/AKT pathway targets and inhibitors.

**Figure 1** summarizes targets of PI3K/AKT pathway and related inhibitors with potential to treat HER2-negative breast cancer.

## DNA Damage Response Inhibitors

DNA damage response (DDR) detecting and repairing damaged genes through a variety of ways is a vital protective mechanism to maintain genome stability and prevent breast cancer. DNA single-strand break (SSB) is mainly repaired by three ways: base excision repair (BER), nucleotide excision repair (NER), and mismatch-repair (MMR), and the more serious DNA double-strand break (DSB) is repaired through two additional pathways: homogeneous recombination (HR) and non-homologous end joining (NHEJ) (44).

HR is an error-free repair process, depending on the availability of homologous DNA templates and mainly playing a role in the G2/M phase of the cell cycle. Although NHEJ is more error-prone compared to HR, it is considered to be the main way of DSB repair and works in all phases of the cell cycle (45).

Mutations in the DDR gene occur in all kinds of breast cancer. Deletion or mutation of BRCA1/2 is present in 10% of patients (46). DNA dependent protein kinase catalytic subunit (DNA-PKcs), a member of the phosphatidyl inositol-3-kinase-like kinase (PIKK) family that is involved in NHEJ and maintains the structural stability of telomeres, is down-regulated in 57% of early breast cancer cases (47). In TNBC, BRCA, non‐BRCA HR, and non‐HR DDR genes have mutations (48), and quite a few proteins involved in DDR including PARP-1 are overexpressed (49).

**Figure 2:**
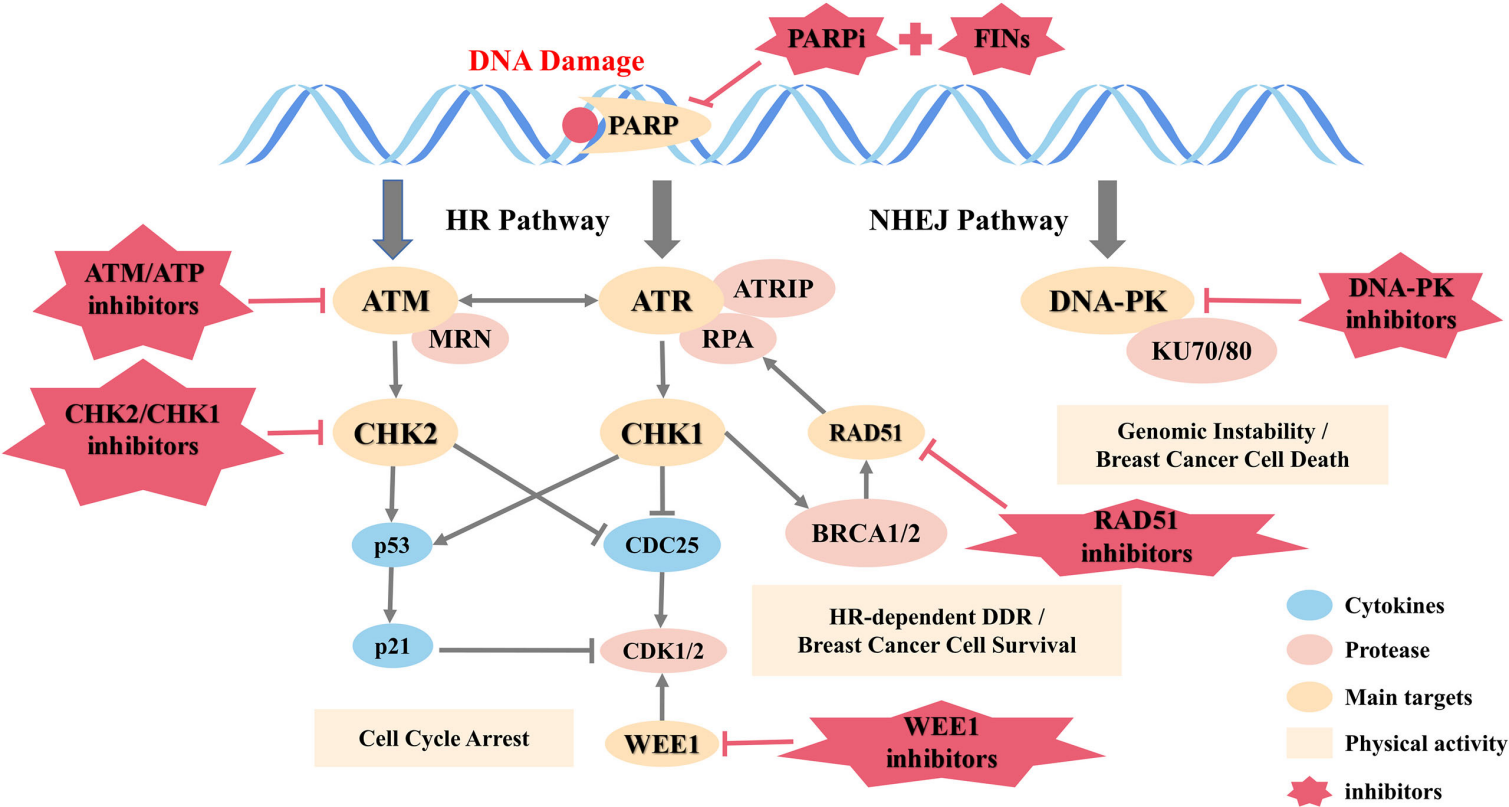
DNA damage response and repair pathways.

A large number of studies have represented that DDR targeted drugs have the potential to treat breast cancer. As shown in **Figure 2**, an overview of the DNA damage response and repair pathways is detailed below.

### PARP Inhibitors

Poly-ADP ribose polymerase (PARP) is located in the nucleus and is a class of enzymes closely related to DDR. It mainly performs a role function in gene transcription, cell differentiation and death (50). In breast cancer cells, inhibiting the function of PARP can interfere with the normal repair of DNA and induce the accumulation of DNA damage, which can be converted into double-strand breaks through replication fork folding, leading to breast cancer cell apoptosis (51). PARP can be cleaved by a variety of caspases, which is regarded as an important indicator of cell apoptosis. Therefore, PARP inhibitors (PARPi) have a satisfactory therapeutic prospect in the treatment of breast cancer. For example, olaparib, niraparib, fluazolepali and pamiparib, currently on the market, has shown good efficacy.

Relevant studies have shown that PARP inhibitors can increase the sensitivity of BCRA1/2 mutant cells (52). BRCA1/2 is an important tumor suppressor gene, with which women take a lifetime risk of breast cancer as high as 85% (53). Accordingly, early studies mainly use PARP inhibitors for breast cancer patients with BCRA1/2 mutations. Olaparib is the first PARP inhibitor approved for the treatment of BRCA-mutated breast cancer. Relevant data in clinical trials shows that olaparib can significantly improve the efficacy of BRCA-mutated and HER2-negative breast cancer patients, and it has fewer side effects and higher safety (54).

PARPi exerts anti-tumor effect by affecting DNA damage repair. It can not only inhibit the catalytic activity of PARP enzyme, but also capture PARP at the site of DNA damage and competitively bind with PARP enzyme. Consequently, it makes PARP1/2 stay in DNA break location, which can prevent DNA repair and promote the conversion of single-strand breaks into double-strand breaks as well. This PARP trapping effect may be more cytotoxic than loss of catalytic activity (55).

Otherwise, iron apoptosis, a type of iron-dependent programmed necrosis, has now been widely recognized as a key factor affecting the occurrence and progression of various cancers (56). Current studies have shown that PARP inhibitors partially achieve the purpose of treatment through ferroptosis. PARPi down-regulates the expression of the cystine transporter SLC7A11 in a p53-dependent manner, thereby causing a decrease in glutathione biosynthesis, promoting lipid peroxidation and leading to ferroptosis (57). As a result, the combined use of ferroptosis inducers (FINs) and PARP inhibitors can enhance the therapeutic effect of breast cancer patients.

### Other DDR Inhibitors

In addition to the above-mentioned PARP inhibitors, currently many drugs that act on other targets of DDR have entered clinical or preclinical trials for the treatment of breast cancer. These inhibitors are often combined with other treatments, especially chemotherapeutic drugs that destroy DNA of breast cancer cells, which can increase the sensitivity of breast cancer cells to drugs, thereby enhancing the efficacy. **Table 2** lists other DDR inhibitors that have the potential to treat breast cancer.

**Table 2:** Other DDR inhibitors that have the potential to treat breast cancer.

Pathway	Target(s)	Name(s)	Potential Targets	Function Study	Reference
Sensors and mediators	ATR	NU6027	↓G2/M phase block and ↓HR	*In vitro*	(44)
Cell cycle checkpoints	CHK1	AZD7762	↓HR	*In vitro* and *in vivo*	(46)
		Prexasertib	↓HR	*In vitro* and *in vivo*	(47)
NHEJ	DNA-PK	LY294002 NU7026 LY294002	↓ATP and ↓DBS repair	*In vitro* and *in vivo*	(50)
NHEJ and PI3K/AKT	DNA-PK and PI3K	KU-0060648	↓ATP and ↓proliferation	*In vitro* and *in vivo*	(51)
HR	RAD51	IBR2	↑RAD51 degradation and ↑apoptosis	*In vitro* and *in vivo*	(52)

↓ - inhibition; ↑ - promotion.

ATM/ATR and MRN complexes play a central role in DDR and cell cycle checkpoints, and these molecules are potential targets for enhancing the sensitivity of breast cancer cells. In recent preclinical experiments, NU6027, a new ATR inhibitor has been shown to effectively inhibit cellular ATR activity (58). NU6027 increases the sensitivity of breast cancer cells to the chemotherapeutic drugs cisplatin and hydroxyurea mainly by attenuating G2/M phase block and reducing HR, which is of great significance for the application of this target inhibitor in the treatment of breast cancer.

Checkpoint kinase (CHK) is a protein kinase involved in cell cycle control. There are currently two subtypes, CHK1 and CHK2. CHK1 is a key regulator of cell cycle and cell survival, regulating S phase, G2/M transition and M phase in normal cell cycle, and participating in initiating DNA checkpoint in DDR to block cell cycle progression. CHK2 is also activated in response to DNA damage and participates in cell cycle arrest. The protein encoded by this gene is a cell cycle checkpoint regulator and a putative tumor suppressor. Studies on CHK1/CHK2 and CDC25 inhibitors are extensive. UCN-01, the first CHK1/CHK2 inhibitor to enter clinical trials, has been shown to have serious side effects, such as symptomatic hypotension and neutropenia, failing to achieve good therapeutic effects (59). AZD7762, a single-target CHK1 inhibitor, can effectively inhibit the proliferation of breast cancer by interrupting HR (60). Prexasertib is a second-generation CHK1 inhibitor, which can play two roles of promoting the post-transformation of BRCA1 and RAD51 proteins and the regulation of transcriptional mediators and inducing HR defects. It is used in combination with PARPi for the treatment of TNBC (61), and the inhibitor has entered early clinical trials (62). CDC25 is a key factor in the activation of cycline-dependent kinases, including CDC25A, CDC25B and CDC25C, which can specifically dephosphorylate the phosphate group on the tyrosine/threonine residues of CDKs, and it is crucial for cell cycle and DDR regulation. CDC25A mainly plays a role in cell G1/S phase transformation, and CDC25B is activated in S phase, which then activates CDK1/cyclin B in cytoplasm, followed by CDC25C activation. This protein is overexpressed in breast cancer and is also considered as a promising new target for breast cancer treatment. However, the current clinical data are limited, and further studies are needed (63).

DNA-PK can connect broken DNA ends and call in other cytokines for repair, being the core target of the NHEJ repair pathway. So far, the most effective inhibition site of DNA-PK is the ATP binding site in the small-molecule targeted kinase domain. Based on this, many small-molecule DNA-PK inhibitors have been designed, such as LY294002, NU7026 and LY294002 (64). However, currently known compounds that specifically inhibit DNA-PK have many limitations in terms of pharmacokinetics, and subsequent studies can improve the efficacy of inhibitors through drug modification and other methods. Ku-0060648, an ATP competition inhibitor inhibiting both DNA-PK and PI3K, restrains breast cancer cell proliferation and enhances the sensitivity of breast cancer cells to chemotherapy drugs doxorubicin and Etoposide (65).

As for HR, there are few inhibitors that directly target HR proteins, but cytokines that indirectly regulate HR, such as RAD51 inhibitors, may also be candidate targets for breast cancer treatment. In the progress of DDR, the non-receptor tyrosine kinase c-Abl activates ATM and phosphorylates RAD51 (66). Oncogenic fusion tyrosine kinases, such as BCR-ABL, TEL-ABL, TEL-JAK2, TEL-PDGFβR, and NPM-AlK are highly expressed in breast cancer cells and promote phosphorylation and expression of RAD51 (67). Therefore, oncogenic fusion tyrosine kinases inhibitors or RAD51 phosphorylation inhibitors can inhibit DDR of breast cancer cells and can be used in combination with chemotherapy drugs to enhance the sensitivity of breast cancer cells. Studies have shown that targeted small-molecule IBR2 can interfere with RAD51, accelerate the degradation of RAD51 protein, thus damage HR, induce apoptosis of breast cancer cells, and provide a new treatment of HER2-negative breast cancer (68).

## Angiogenesis Inhibitors

Neovascularization is the basis of growth and metastasis of breast cancer, and targeted inhibition of angiogenesis can effectively inhibit the growth of breast cancer. Among various factors which have the regulatory function of newborn angiogenesis, vascular endothelial growth factor (VEGF), one of the most effective factors, can bind to VEGF receptor (VEGFR), like VEGFR-1 and VEGFR-2, stimulating the proliferation and migration of endothelial cells through specific signaling transduction pathways, thereby promoting the formation of neovascularization (69, 70). Among them, VEGFR-2 regulates the differentiation, migration and proliferation of endothelial cells, while VEGFR-1 regulates the maintenance of blood vessels in the late developmental stage (71). In addition, a naturally occurring soluble form of VEGFR-1 (SVEGFR-1) is an important inhibiting factor of angiogenesis mediated by VEGF, which can inhibit proliferation of endothelial cells due to VEGF (72). Therefore, VEGF and VEGFR and their associated downstream signaling pathways may serve as targets for HER2-negative breast cancer drugs.

At present, many angiogenic inhibitors targeting VEGF/VEGFR have been marketed and played clinical roles, such as bevacizumab, which has achieved satisfactory results in combination with taxanes (73). However, studies have shown that there is a significant correlation between VEGF and HER2 expression (74), and such targeted drugs are not suitable for patients with HER2-negative breast cancer. Considering this issue, the development of angiogenesis inhibitors for HER2-negative breast cancer has become a hot spot of new drug research and development, and many related drugs have entered the stage of clinical research.

### Tyrosine Kinase Inhibitors

Tyrosine kinase inhibitors (TKIs) act on vascular endothelial growth factor receptor (VEGFR), platelet-derived growth factor receptor (PDGFR), stem cell factor receptor, colony-stimulating factor-1 receptor and fms-like tyrosine kinase-3 (75–79), inhibit angiogenesis from multiple targets and effectively inhibit the growth of tumor.

Although TKIs are only applied to a small number of solid tumors at present and have not shown satisfactory efficacy in the application of breast cancer, existing studies have shown that VEGF and PDGFR are significantly correlated with the prognosis of breast cancer (80, 81), and TKIs have great potential in the treatment of breast cancer.

In preclinical studies, Sorafenib and Sunitinib, two drugs of TKIs acting on various targets and inhibiting multiple pathways, failed to achieve good therapeutic effect in the treatment of breast cancer by using single drug, while the combination of drugs led to serious adverse reactions (82). In addition, due to the “off-target” effect, traditional vascular targeting drugs can induce vasculogenic mimicry (VM), which induces endothelial cells to form vascular-like channels responsible for the supply of blood, nutrients, and oxygen, resulting in drug resistance (83–85). In the context of poor efficacy of most TKIs, apatinib, a kind of TKIs that only highly binds VEGFR-2 target, has shown good efficacy and tolerability in patients with advanced TNBC (86), which can be further used in combination with other chemotherapy drugs to verify the feasibility of TKIs in the treatment of HER2-negative breast cancer.

### Antiangiogenic Effects of Other Drugs

Endocrine therapy for breast cancer is mostly used in ER positive patients, and tamoxifen is the most commonly used anti-estrogen therapy. Existing data indicate that a variety of estrogen hormones, such as estradiol and progesterone, increase the expression level of VEGF in breast cancer (87, 88), while tamoxifen can inhibit the secretion of VEGF (89) and reduce the density of vascular endothelial cells in breast cancer by more than 50%, the mechanism of which is related to the regulation of the expression ratio of VEGF and sVEGFR-1 (90).

In addition, there is a crossover between the downstream signaling pathway of ER and VEGF signaling pathway in breast cancer (91), which provides a theoretical basis for clinical endocrine therapy combined with other angiogenesis inhibitors.

### Endostatin

Endostatin (ES) is a kind of angiogenesis inhibitor extracted from the culture medium of EOMA cells, and the cells are derived from mice with hemangioendothelioma. ES has the strongest and broadest curative effect in suppressing tumors. Endostar, a modified recombinant human endostatin, was first developed as an anti-tumor agent in 2003 and is currently being fully studied in combination with chemotherapy for advanced breast cancer. Studies have shown that Endostar can decrease the expression of MMP-2 and MMP-9 in TNBC cells, inhibit the phosphorylation of ERK1/2, and significantly prevent the proliferation and migration of breast cancer cells (92). For patients with HER2-negative breast cancer, especially patients with TNBC, Endostar combined with chemotherapy drugs can be actively used for treatment.

## Cell Cycle Inhibitors

Cell cycle is the basic process of cell life activities and is regulated by multiple signaling pathways such as PI3K/AKT signaling and MAPK signaling. Disturbance of cell cycle is the most significant mechanism of breast cancer. And researching on new drugs that inhibit the cell cycle can provide new ideas and methods for breast cancer treatment.

**Figure 3:**
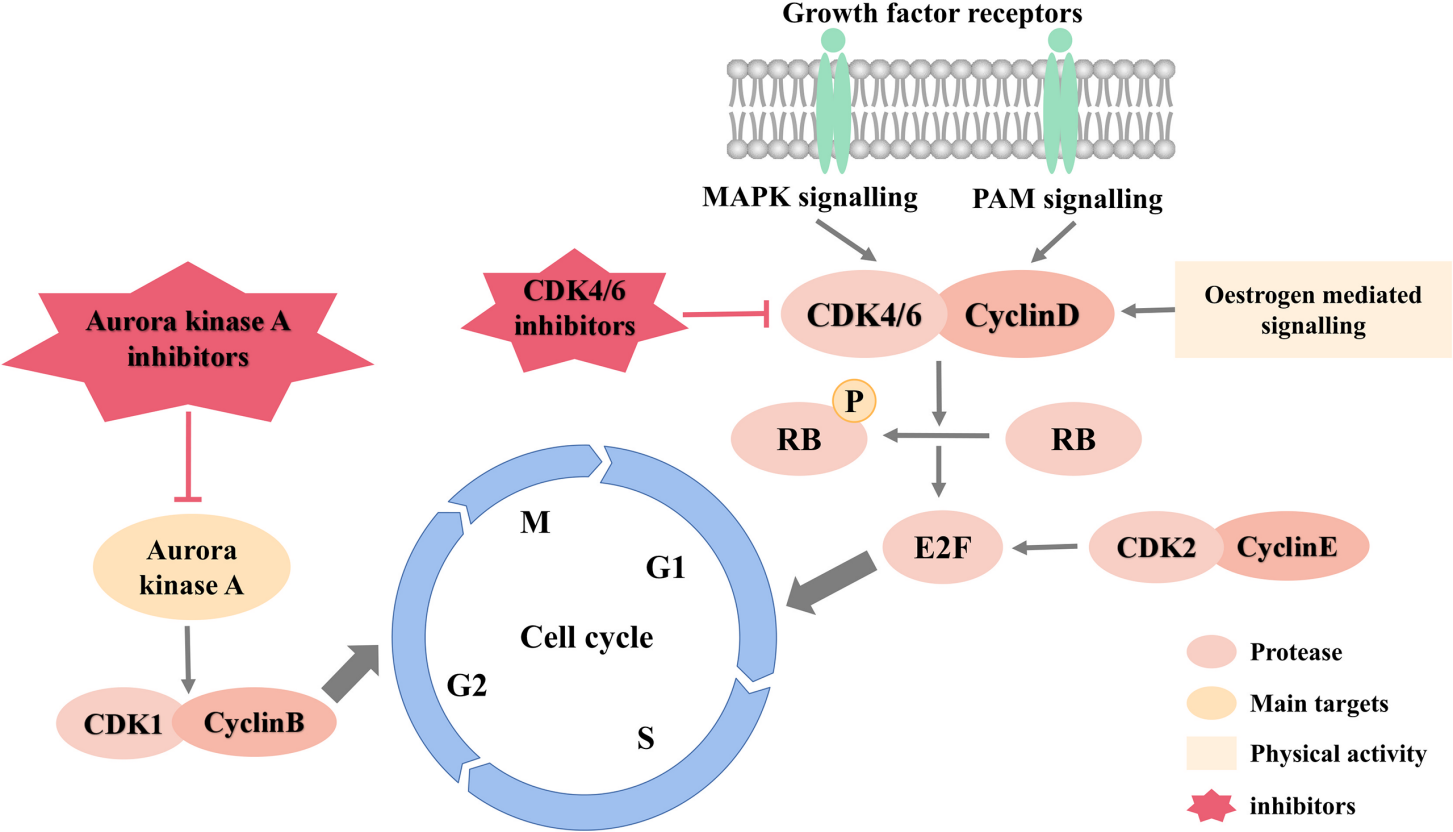
Cell cycle signaling pathways and inhibitors.

**Figure 3** is an overview of cell cycle signaling pathways and inhibitors.

### CDK Inhibitors

Cyclin dependent kinase (CDK), a key kinase involved in regulating the cell cycle, can be combined with cyclin to form an active heterodimer, which plays an essential role in the initiation of cell cycle and the regulation of transformation in every period. At present, more than 20 different kinds of CDKs have been reported. Among them, CDK1, CDK2, CDK4 and CDK6 participate in cell cycle regulation. In breast cancer cells, cyclin is overexpressed or overactivated, CDKI activity is inhibited, and the continuous activation of upstream fission signalings leads to the deregulation of CDK activity, which directly or indirectly causes uncontrolled cell proliferation and genome instability, resulting in the occurrence and development of breast cancer (93). Since CDK activity is necessary for the growth of breast cancer cells, CDK has been considered a good target for breast cancer drugs for a long time. Currently, dozens of CDK inhibitors are undergoing clinical or preclinical research (94). According to their different mechanisms of action, these inhibitors can be divided into ATP competitive and non-competitive inhibitors (95).

ATP-competitive CDK inhibitors play an inhibitory effect by mimicking the ATP structure and binding to CDK protein (96), and the development of these CDK inhibitors is progressing relatively smoothly. The first-generation inhibitors, such as flavopiridol, roscovitine, UNC-01, etc., due to the lack of selectivity for different types of CDK and serious side effects in the clinical, their development was stopped (97, 98). On the contrary, the second generation of CDK inhibitors show better anti-tumor activity and selectivity, especially those targeting CDK4/6, like palbociclib, ribociclib, abemaciclib, etc., which can inhibit RB phosphorylation and block the cell cycle in G1 phase, preventing the proliferation of breast cancer (99, 100). Clinical trials have shown that it has good efficacy in the treatment of metastatic breast cancer with HR+ and HER2- when combined with endocrine therapy (101).

CDK4/6 inhibitors are currently widely used in the treatment of breast cancer but only used in patients with ERα-positive breast cancer because of their frequent overexpression of cyclin D. At the same time, most HER2-negative breast cancers have RB1 mutations and/or loss, limiting the use of CDK4/6 inhibitors (102). Fortunately, the expression of androgen receptor (AR) is positively correlated with RB, which promotes cyclin D activation (103, 104), suggesting that CDK4/6 inhibitors have great potential in the treatment of HER2-negative breast cancer with AR-positive. In fact, related studies have combined CDK4/6 inhibitor abemaciclib with seviteronel, which targets androgen biosynthesis and AR activity, and showed synergistic effects in AR-positive TNBC models (105). Therefore, cell cycle inhibitors can be used in combination with AR targeted drugs to treat AR-positive TNBC.

The development of non-ATP-competitive CDK inhibitors has been relatively slow. Such inhibitors mainly include peptides and synthetic small molecules, which mimic the endogenous CDK inhibitors, such as p21, p27, p25, etc., to exert their inhibitory effects (106, 107). Various novel methods for interference with CDK and the cyclin complex have emerged, such as preventing substrate recognition, targeting essential protein-protein interactions, targeting residues necessary for conformational changes, etc. (108, 109), and thus substrate competition inhibitors and heterogeneous inhibitors are developed (95). Substrate competition inhibitors mainly prevent the binding of CDK and cyclin, thereby inhibiting CDK activity. At present, such inhibitors have been expanded to develop better drug-like inhibitors of CDK2 polypeptide analogs (110). The heterogeneous inhibitor usually binds to the vicinity of the ATP binding site, which interferes with the conformational transition of the enzyme and has good selectivity (111). Heterogeneous inhibitors have been successfully applied in the research and development of ABL/P38 and MEK1 inhibitors. It is a promising strategy for the development of CDK inhibitors and has great potential in the treatment of breast cancer (112).

### Aurora Kinase Inhibitors

Aurora kinase is a kind of mitotic kinase, mainly including aurora A, aurora B and aurora C. The gene of aurora A is located in the active amplification segment of the chromosome, which is amplified and overexpressed in breast cancer cells (113). Studies have found that aurora A can interact with tumor suppressor gene p53 (114) and also bind and phosphorylate SRCA1, resulting in BRCA1 dysfunction (115), which is closely related to the occurrence and development of breast cancer. Loss or inhibition of aurora A will lead to the appearance of unipolar or multistage spindle and the failure of centrosome separation, inhibiting growth and proliferation of cells (116, 117). The main functions of aurora B are to promote chromatin condensation and to monitor cell cycle checkpoints, having a regulating effect in various stages of cell mitosis (118). Currently, as a potential target for breast cancer, the aurora kinase has attracted extensive attention. An increasing number of aurora kinase inhibitors have been developed, and some of them have entered the stage of clinical trials.

ZM-447439 and hesperidin are both aurora B kinase inhibitors. The former can increase the number of apoptotic cells and the formation of polyploidy which depends on p53 (119), while the latter inhibits the phosphorylation of histone H3, causes chromosome separation and abnormal cytokinesis, and leads to polyploidy, which has great potential in inhibiting tumor cell metastasis, angiogenesis and chemotherapy (120). At present, a lot of novel aurora kinase inhibitors only have effects on aurora A, such as MLN-8237, which has been found to promote the apoptosis and autophagy of breast cancer cells by regulating the p38 MAPK/Akt/mTOR pathway and has entered the second phase of clinical trials. It has shown great potential in the treatment of HER2-negative breast cancer (121).

Because the aurora kinase is highly expressed in breast cancer cells and less active in normal or resting cells, aurora kinase inhibitors are highly selective for breast cancer cells. However, the current research on the targeting of aurora kinase inhibitors is almost all about the competitive inhibition of ATP binding sites, resulting in poor selectivity of inhibitors and susceptibility to drug resistance. Therefore, the development of other targets such as ATP non-competitive aurora kinase inhibitors or substrate competitive inhibitors may help overcome the current difficulties encountered in aurora kinase inhibitors, and further contribute to the application of such inhibitors in the treatment of breast cancer.

### Histone Deacetylase Inhibitors

Histone deacetylase (HDAC) can affect the structural modification of chromosome and gene transcription and expression, playing a pivotal role in the acetylation of non-histones and histones (122). HDAC1 is one of the members of all histone deacetylases most closely related to breast cancer and plays a crucial part in the occurrence and metastasis of breast cancer (123). The high expression of HDAC1 in breast cancer cells leads to high deacetylation of core histones and chromosome condensation, inhibiting the transcription of related genes and inducing the occurrence of breast cancer (124). Additionally, it can also regulate the apoptosis of breast cancer cells through a variety of ways (125) and inhibiting the activity of HDAC can effectively restrain the proliferation of breast cancer cells.

Since the first potent HDAC inhibitors emerged, there have been five HDAC inhibitors approved for use as oncology chemotherapy agents, and many others are in clinical or preclinical trials (126). Among them, SAHA appeared earlier and has been widely studied. It can regulate the function of the promoter of p21WAF1/CIP1- a cell cycle suppressor protein, obviously induce its expression, and block the cell cycle in the S phase to play an anti-tumor effect (127). Recent research has shown that the sensitivity of TNBC to HDAC inhibitors can be enhanced when used in combination with leukemia inhibitory factor receptor (LIFRα) inhibitors, and such inhibitors have a bright prospect in the combination therapy of HER2-negative breast cancer (128).

## AR Inhibitors

An important feature of breast cancer is hormone dependence, especially sex hormones. Androgen receptor (AR), like ER and PR, is a member of the superfamily of nuclear receptors, which mainly exists in the target nucleus and belongs to steroid receptors. It is highly expressed in breast cancer and has long been an important indicator of breast cancer diagnosis (129). The expression of AR is closely related to various subtypes of breast cancer. Choi et al. showed that the expression of AR was positively correlated with histological grade and negatively correlated with OS and disease-free survival (DFS), indicating that AR is an indicator of poor prognosis (130). In recent years, many studies have confirmed that AR plays an important role in the occurrence and development of breast cancer, but this role is not directly induced by AR independent signaling pathway, but closely related to multiple pathways, such as HER-2, wnt, ERα and MAPK (131, 132). Studying AR signaling pathway can not only reflect the severity of the disease, but also provide ideas for the treatment of breast cancer (129).

### AR Targeted Inhibitors

Currently on the market, AR targeted inhibitors are mainly bicaluramide and azaluramide, both of which are non-steroidal AR inhibitors. They are mainly used for the treatment of prostate, but many recent studies have shown that they also play an important role in the treatment of breast cancer.

Enzarumide is a powerful inducer of cytochrome P4503A4, which can not only improve the metabolism of aromatase inhibitors, but also reduce the nuclear localization of AR. Schwartzberg et al. combined the AR inhibitor Enzaruamide with endocrine therapy to treat breast cancer and found that it had a good effect (133). Bicaluamide can effectively control androgen level by combining with AR, thus promoting breast cancer cell death (132).

Since AR is expressed in all subtypes of breast cancer, especially TNBC, which lacks a clear target, AR is expected to become a potential target for breast cancer treatment.

### Androgen Synthesis Inhibitors

CYPC17 is an important androgen synthase, and abiraterone is a CYPC17 inhibitor, which can effectively control the synthesis of androgen and estrogen, thus reducing the resistance to hormone therapy for breast cancer (134). Although abiraterone is currently clinically used in the treatment of prostate cancer, with the in-depth study of AR expression in breast cancer, it has a theoretical prospect of application in the treatment of breast cancer. It is hoped that further studies will be conducted to confirm its effect on HER2-negative breast cancer.

## Targeting Strategies for BCSCs

Breast cancer stem cells (BCSCs) are very few stem cell subgroups with ability of self-renewal and multidirectional differentiation in breast cancer cells and play an important role the recurrence, metastasis and drug resistance of breast cancer (135, 136). Relevant studies have shown that BCSCs can activate the STAT3 signaling pathway or the Notch-1-PTEN-ERK1/2 signaling pathway under the stimulation of certain transcription factors and inflammatory factors to promote breast cancer recurrence (137, 138). At the same time, BCSCs can prompt epithelial-mesenchymal transformation (EMT) of breast cancer cells through TGF-β signaling transduction (139), thus inducing the invasion and migration of breast cancer and reducing the survival rate of patients. Therefore, it is of great clinical significance to further understand CSCs and develop relevant targeted drugs for the treatment of breast cancer.

Conventional radiotherapy or chemotherapy can only target BCSCs with active proliferation and has no killing effect on BCSCs that are in the resting state, while residual BCSCs often cause breast cancer recurrence and metastasis when activated by appropriate signalings, and targeted elimination of BCSCs may be an effective strategy to improve the prognosis of breast cancer patients (140). Presently, targeting strategies for BCSCs mainly include cell targeted therapy, gene targeted therapy and nano-delivery targeted therapy, etc.

### Cell Targeted Therapy

Mesenchymal stem cells (MSCs) have become a promising therapeutic agent for targeting BCSCs in the cell targeted therapy of breast cancer (141). MSCs have the characteristics of multi-differentiation potential, low immunogenicity, and homing to tumor tissues. They can be targeted to migrate to breast cancer sites under action of multiple factors and are an important part of breast cancer microenvironment (142, 143). Mandal et al. showed that MSCs can inhibit the proliferation of BCSCs, prevent the formation of EMT and reduce angiogenesis (144), which has a good inhibitory effect on the proliferation and metastasis of breast cancer. In addition, due to the low immunogenicity and the inherent tumor-homing ability, MSCs can be used to load novel nano-chemotherapy drugs to achieve the purpose of targeted delivery to breast cancer cells, providing a new solution for HER2-negative breast cancer targeted drugs (145).

In addition to MSCs, long non-coding RNA (lncRNA) and microRNA (miRNA) can also regulate the proliferation of CSCs, which has important value in the treatment of HER2-negative breast cancer (146, 147). Studies have shown that lncRNA CCAT2 is overexpressed in TNBC and BCSCs. It promotes the occurrence and development of breast cancer by up-regulating the expression of OCT4-PG1 and activating the Notch signaling pathway (148). In metastatic breast cancer tissues, it has been found that the expression level of miR-200c is decreased miR-300c is increased, which may be a sign of enrichment of BCSCs in patients (149). Therefore, targeting lncRNA or miRNA may contribute to developing new treatments for BCSCs.

### Nano-Delivery Targeted Therapy

Recently, it has been discovered that molecular-oriented nanotechnology can be applied to the development of BCSCs targeted drugs, which can effectively control drug delivery and release. Not only can it improve the absorption of drugs by CSCs, but also increase the retention and release of drugs in breast cancer cells. The mechanism is to down-regulate the expression of Sox2 and ABCG2, reduce the ratio of BCSCs, and enhance drug retention and sustained release. In addition, using it in combination with taxanes can increase the sensitivity of breast cancer cells to drugs (150).

The application of RNA nanotechnology to deliver anti-miRNA has become a new technology for TNBC therapy. These therapeutic RNA nanoparticles bind to the CD133 receptor to inhibit the expression of miR21 and up-regulate the expression of downstream tumor suppressor genes PTEN and PDCD4, which can significantly inhibit the proliferation of TNBC cells (151).

The development of nanotechnology provides a new and effective method to deliver drugs for the treatment of breast cancer. Drug efficacy can be enhanced and side effects can be reduced by combining with existing breast cancer treatments, which is indispensable for the treatment and prognosis of breast cancer.

## Conclusion

Breast cancer is the most frequently diagnosed cancer and the main cause of death from cancer in women. With the development of molecular biology and genomics, molecular targeted therapy has become one of the most active areas in the treatment research of breast cancer, and it has also achieved remarkable achievements. At present, targeted therapy has become a brand new biological treatment method in addition to the four traditional treatment modes of surgery, radiotherapy, chemotherapy and endocrine therapy.

However, today’s targeted therapy drugs mainly target HER2-positive breast cancer. The advent of trastuzumab, pertuzumab, lapatinib and other drugs has significantly improved the prognosis of patients with HER2-positive breast cancer (152). The drugs approved for the treatment of HER2-negative breast cancer and related information are listed in **Table 3**. It can be seen that targeted drugs for HER2-negative breast cancer, especially TNBC, are relatively less selective, and there are still many problems waiting to be solved. For example, the efficacy of single-agent therapy is not satisfactory, the price is expensive, which increases the economic burden of patients, and the target selectivity is so low that it is prone to high toxicity (153). Therefore, it is necessary to continuously develop and research more accurate and efficient new drugs that can also reverse drug resistance.

**Table 3:** Approved for nearly a decade to treat HER2-negative breast cancer.

Pathway	Name(s)	Target(s)	Time	Indication	ADR
PI3K	Alpelisib	PI3Kα	2019	HR+/HER2- advanced or metastatic breast cancer with PIK3CA mutations	Hyperglycemia, pneumonia, rash, diarrhea, embryo-infantile toxicity, and other toxicity, etc.
mTOR	Everolimus	FKBP12	2012	HR+/HER2-advanced breast cancer in combination with Aremassin	Pharyngitis, lack of appetite, diarrhea, fatigue, rash, infection and oral ulcers, etc.
DDR	Talaziparib	PARP	2018	HER2-/gBRCAm+ locally advanced or metastatic breast cancer	Fatigue, anemia, nausea, neutropenia, headache, vomiting, hair loss, diarrhea, loss of appetite and embryo-fetal toxicity, etc.
DDR	Olaparib	PARP-1 and PARP-2	2018	BRCA+/HER2- metastatic breast cancer	Anemia, nausea, fatigue, vomiting, taste disorders, dyspepsia, headache, loss of appetite, arthralgia, myalgia, rash and abdominal pain, etc.
The cell cycle	Palbociclib	CDK4/6	2015	HR+/HER2- advanced or metastatic breast cancer	Neutropenia, leukopenia, fatigue, anemia, upper respiratory tract infection, nausea, diarrhea, anorexia, vomiting, fatigue, etc.
The cell cycle	Ribociclib	CDK4/6	2017	Initial endocrine therapy in HR+/HER2- advanced or metastatic breast cancer	Leukopenia, nausea, fatigue, diarrhea, hair loss, vomiting, constipation, headache, etc.
The cell cycle	Abemaciclib	CDK4/6	2018	HR+/HER2- advanced or metastatic breast cancer	Diarrhea, neutropenia, nausea, infection, fatigue, anemia, leukopenia, loss of appetite, vomiting, headache, etc.
TROP-2	Sacituzumab govitecan-hziy	Topoisomerase	2020	Metastatic TNBC that has been treated with at least two therapies	Tired, hair loss, constipation, rash, decreased appetite, stomachache, neutropenia, severe diarrhea, anaphylaxis, nausea, vomiting, etc.
Hormone therapy	Enzalutamid	Androgen	2012	Advanced TNBC	Fatigue, external edema, myomyalgia, headache, muscle weakness, vertigo, insomnia, anxiety and hypertension

The most widely used targeted drugs for HER2-negative breast cancer are DDR inhibitors, especially PARP inhibitors, which have shown good efficacy in treatment of TNBC (54). In addition, as most chemotherapy drugs work by destroying DNA of breast cancer cells, DDR inhibitors can not only inhibit breast cancer cells, but also improve their sensitivity to such chemotherapy drugs. The combination of the two drugs can achieve twice the result with half the effort.

Inhibitors targeting the PI3K/AKT pathway, angiogenesis and blocking of the cell cycle are already under development. Studies have shown that the therapeutic effect of inhibitors targeting the PI3K/AKT pathway is more obvious in patients with high levels of phosphorylated AKT, loss of PTEN protein and mutations of PTEN or PIK3CA genes. Unfortunately, these inhibitors are obstructed by the high incidence of adverse reactions such as diarrhea, neutropenia, and pneumonia (38). Currently, angiogenesis inhibitors are mainly used in patients with HER2-positive breast cancer, and their efficacy in patients with HER2-negative breast cancer should be further studied. CDK 4/6 inhibitors have brought new hope for breast cancer treatment. Multiple studies have confirmed that this inhibitor combined with endocrine therapy can significantly improve the survival of patients with HR-positive and HER2-negative advanced breast cancer (154–156). Nevertheless, many current studies attach importance to improving the resistance of breast cancer cells to CDK 4/6 inhibitors. It is hoped that these inhibitors can be further used in the treatment of breast cancer (157, 158).

Due to the high selectivity and low toxicity of stem cells, the therapy targeting stem cells has become a hot topic in recent years. MSCs, lncRNA and miRNA in HER2-negative breast cancer treatment have important application value (159), in addition, the new technology such as nanometers provides a new drug delivery system, which can improve drug efficacy and reduce drug side effects at the same time.

Factly, all drugs for the treatment of HER2-negative breast cancer do not only act on a single pathway, but rather, a single drug can regulate multiple pathways (160), such as the recently widely studied effects of statins on breast cancer.

Statins are HMG-CoA reductase inhibitors, which are often used to reduce cholesterol levels and prevent coronary heart disease in clinical practice (161). In recent years, it has been found that statins have significant efficacy in the treatment of tumors, especially for ER-positive breast cancer (162, 163). A large number of statins, such as simvastatin, lovastatin and fluvastatin, can inhibit the proliferation and migration of breast cancer. The mechanisms related to their therapeutic effects include inhibition of PI3K/Akt and PPTG1 signaling pathways, activation of LKB1-AMPK-P38MAPK-p53-survivin cascade resulting in cell death, and increased caspase-3-mediated vimentin hydrolysis leading to the death of breast cancer cells (164–167), indicating that it achieves the purpose of breast cancer treatment by regulating multiple signaling pathways.

With the rapid development of modern technology and biomedicine, the treatment concept of breast cancer is constantly updated. In recent years, breast cancer is considered as a systemic disease, and neoadjuvant chemotherapy has also been included as an important part of the treatment of HER2-negative breast cancer (168). At the same time, the treatment and drugs for HER2-negative breast cancer are also undergoing comprehensive innovation, with more influencing factors being included in the development of new drugs (160, 169). In addition, more studies are promoting the clinical treatment towards the direction of “precision”.

Precision medicine is based on personal genomic information, combined with proteome, metabolome and other relevant internal environment information, to tailor the best treatment plan for patients, in order to maximize the therapeutic effect and minimize side effects. The study of gene expression profiles of various subtypes of breast cancer and the expression of specific target molecules, as well as the discovery of specific targeted therapeutic measures, can further realize the goal of “personalized medicine”.

In HER2-negative breast cancer treatment, more studies on the combination of targeted therapies with other therapies need to be carried out, in order to further study the effectiveness, safety and economics of the combined application of targeted drugs and chemotherapy drugs, so as to maximize the efficacy of targeted breast cancer therapy, which may become a new direction for the treatment of breast cancer patients in the future.

## Author Contributions

This review was conceptualized by all the authors. JA: project conception, original draft preparation. FP, CP, and XX edited the manuscript. FP: funding administration. FP, CP, and XX: manuscript revision. All authors contributed to the article and approved the submitted version.

## Funding

This research was funded by the National Natural Science Foundation of China (82003879), Youth Talent Promotion Project of China Association for Science and Technology (CACM-2020-QNRC1-01), the Key Project of Science and Technology Department of Sichuan Province (No. 2020YFS0053; 2021YFS0044), and the Open Research Fund of Chengdu University of Traditional Chinese Medicine Key Laboratory of Systematic Research of Distinctive Chinese Medicine Resources in Southwest China.

## Conflict of Interest

The authors declare that the research was conducted in the absence of any commercial or financial relationships that could be construed as a potential conflict of interest.

## Publisher’s Note

All claims expressed in this article are solely those of the authors and do not necessarily represent those of their affiliated organizations, or those of the publisher, the editors and the reviewers. Any product that may be evaluated in this article, or claim that may be made by its manufacturer, is not guaranteed or endorsed by the publisher.
